# ﻿*Elsholtziazhongyangii* (Lamiaceae), a new species from Sichuan, China

**DOI:** 10.3897/phytokeys.193.80327

**Published:** 2022-03-22

**Authors:** Xin-Jie Jin, Yue Huang, Yu-Kun Wei, Qing Ma, Lu-Xian Liu, Zhi-Xi Fu, Gui-Fang Wu, Yong-Hua Zhang, Pan Li

**Affiliations:** 1 College of Life and Environmental Science, Wenzhou University, Wenzhou, 325035, China Wenzhou University Wenzhou China; 2 Eastern China Conservation Centre for Wild Endangered Plant Resources, Shanghai Chenshan Botanical Garden, Shanghai 201602, China Eastern China Conservation Centre for Wild Endangered Plant Resources, Shanghai Chenshan Botanical Garden Shanghai China; 3 College of Biology and Environmental Engineering, Zhejiang Shuren University, Hangzhou 310015, China Zhejiang Shuren University Hangzhou China; 4 Key Laboratory of Plant Stress Biology, School of Life Sciences, Henan University, Kaifeng 475001, China Henan University Kaifeng China; 5 College of Life Sciences, Sichuan Normal University, Chengdu 610101, China Sichuan Normal University Chengdu China; 6 Laboratory of Systematic & Evolutionary Botany and Biodiversity, College of Life Sciences, Zhejiang University, Hangzhou 310058, China Zhejiang University Hangzhou China

**Keywords:** Aromatic plant, Elsholtzieae, phylogeny, traditional medicinal herbs, taxonomy

## Abstract

*Elsholtziazhongyangii* (Lamiaceae), a new species from Sichuan Province, China, is described and illustrated. The new species is morphologically similar to E.feddeif.feddei, but it can be easily distinguished from E.feddeif.feddei by smaller corolla (3.2–3.5 mm vs. 4.5–5.3 mm), bract indumentum (glabrous, except margin ciliate vs. villous, especially on veins abaxially, glabrous adaxially) and bract stalked (ca. 1.2 mm vs. sessile). Phylogenetic analyses, based on two nuclear ribosomal (ETS, ITS) and five plastid (*rbc*L, *mat*K, *trn*L-F, *ycf*1, *ycf*1-*rps*15) regions, confirmed that the new species formed a monophyletic clade with robust support. The new species is currently known from western Sichuan.

## ﻿Introduction

*Elsholtzia* Willdenow (Lamiaceae) is a member of the tribe Elsholtzieae (Harley et al. 2004). The genus is characterised by verticillasters in continuous or interrupted spikes or capitula, compact spikes cylindric or secund, and two-lipped corolla (Huang 1977) (Huang 1977). *Elsholtzia* is mainly distributed in East Asia, with 35 species found in China (Huang 1977; Li and Hedge 1994; Harley et al. 2004; Pu et al. 2012; Xiang and Liu 2012). Many species of *Elsholtzia* are important plant resources used both as medicine and flavouring. More than 60% of the Chinese species have been widely used as traditional medicinal herbs, nectar source plants, vegetables and spices (Huang 1977; Li and Hedge 1994; Editorial Board of Zhong Hua Ben Cao 1999).

During our botanical expedition to Yajiang County, Sichuan Province in September 2012, we discovered an unknown species of *Elsholtzia*. It is similar to Elsholtziafeddeif.feddei in calyx (villous, with two long and three short teeth with spinescent apices) and leaf apices acute, but differs from the latter by its smaller corolla (3.2–3.5 mm vs. 4.5–5.3 mm), bract glabrous, with a ciliate margin (vs. villous, especially on veins abaxially, glabrous adaxially) and bract stalked (ca. 1.2 mm vs. sessile). After carefully checking specimens and literature, together with evidence from molecular phylogenetic analyses, based on combined nrDNA (ETS, ITS) and combined ptDNA (*rbc*L, *mat*K, *trn*L-F, *ycf*1, *ycf*1-*rps*15) datasets, we demonstrated that it is, indeed, a new species which is described and illustrated here.

## ﻿Materials and methods

### ﻿Morphological study

The morphological characters were examined, based on the living plants and specimens. *Elsholtzia* specimens, collected from Sichuan, were checked in the Herbaria of **CDBI**, **CDCM**, **CQNW**, **HITBC**, **HNWP**, **HZU**, **IBK**, **IBSC**, **KUN**, **NAS**, **PE**, **SM**, **SZ**, **TIE**, **WCSBG**, **WUK** and **WZUH** (acronyms as in Thiers 2021).

### ﻿Taxon sampling and molecular analyses

**Figure 1.**
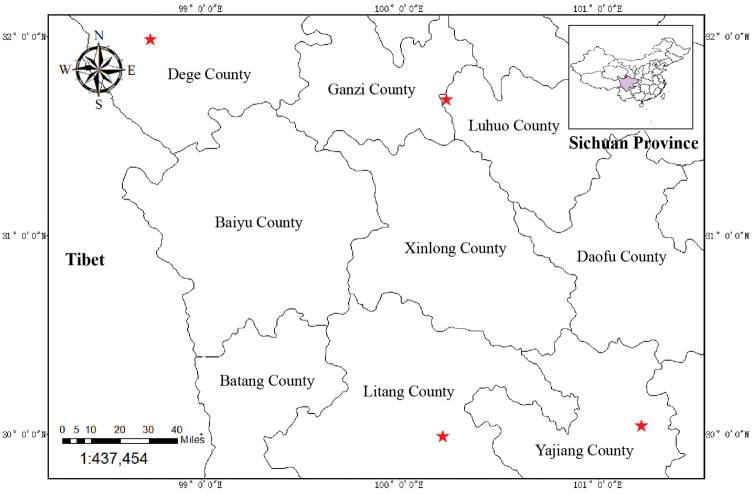
Distribution of *Elsholtziazhongyangii* Pan Li & X.J. Jin. The red stars indicate the recorded localities of *E.zhongyangii*.

A total of 48 individuals of *E.zhongyangii* were collected from Yajiang County, Sichuan Province, China from September 2012 to December 2021 (Fig. 1). Voucher specimens were deposited in Wenzhou University (**WZUH**). The nuclear (ETS, ITS) and plastid (*rbc*L, *mat*K, *trn*L-F, *ycf*1, *ycf*1-*rps*15) regions were used for reconstructing the phylogeny of the new species and its related taxa (Li et al. 2017). A total of 20 individuals, representing 16 species of *Elsholtzia*, were sampled, with *Elsholtziadensa* Benth. as the outgroup. The GenBank accession numbers are listed in Suppl. material 1. Most sequences were downloaded from GenBank, except for the new species, which was newly sequenced in the present study.

Two samples of the new species were sequenced using the genome skimming approach, then 14 sequences [each sample has two nrDNA (ETS, ITS) and five ptDNA (*rbc*L, *mat*K, *trn*L-F, *ycf*1, *ycf*1-*rps*15) sequences as listed in Suppl. material 1] were mined for the phylogenetic analyses. Genomic DNA was extracted from approximately 20 mg of silica-gel-dried leaves using DNA Plantzol Reagent (Hangzhou Lifefeng Biotechnology Co., Ltd.) according to the manufacturer’s protocol. For each species, genomic DNA (m > 1 μg, c > 12.5 ng/μl) was sheared (yielding < 800 bp fragments) and the quality of fragmentation was checked on an Agilent 2100 Bioanalyzer (Agilent Technologies). Preparation of short-insert (350 bp) paired-end libraries and sequencing were performed by the Beijing Genomics Institute (Shenzhen, China). These samples were pooled with others and run in a single lane of an Illumina HiSeq XTEN with a read length of 150 bp. For the two samples of the new species, we used the GetOrganelle pipeline (Jin et al. 2020) for de novo assembly of plant plastome and nuclear ribosomal RNA (ETS-18S-ITS1-5.8S-ITS2-26S-ETS). The nuclear (ETS, ITS) and chloroplast (*rbc*L, *mat*K, *trn*L-F, *ycf*1, *ycf*1-*rps*15) segments were extracted from these assembled genomic sequences.

Maximum Likelihood (ML) analyses were performed in IQtree v.1.6.12 (Nguyen et al. 2015). The best-fitting substitution models (GTR+F+G4 for nrDNA and TVM+F+G4 for ptDNA) were selected by ModelFinder (Chernomor et al. 2016; Kalyaanamoorthy et al. 2017) according to the Bayesian Information Criterion (BIC). An ultrafast bootstrap (UFB) (Bui et al. 2013) of 1000 replications and the SH-aLRT test were used in the analysis to assess branch supports. Bayesian Inference analyses (BI) were conducted in MrBayes 3.2.6 online interface (Miller et al. 2010). MrModeltest 2.3 (Nylander 2004) was used to determine the appropriate DNA substitution model using the Akaike Information Criterion (AIC) and the results indicated that GTR+G (both for nrDNA and ptDNA) is the best-fit model. Four (one cold and three hot) simultaneous Markov chains were run for one million generations with sampling every 1000^th^ generation until the average deviation of split frequencies fell below 0.01. The posterior distribution of trees was summarised by the > 50% majority rule consensus tree after discarding the first 25% of samples as burn-in.

Incongruences amongst different datasets (combined ptDNA dataset, combined nrDNA dataset) were explored through visual comparison of tree topologies and support values. Hard incongruence was defined as BS ≥ 80% and/or PP ≥ 0.95 (Pelser et al. 2010).

## ﻿Results

### ﻿Morphological comparison

Detailed morphological comparisons between the new species and four other sympatric or morphologically similar taxa are summarised in Table 1. In morphology, the putative new species is most similar to Elsholtziafeddeif.feddei, sharing features, such as two long and three short calyx teeth with spinescent apices, white villous outside of the calyx, acute leaves apex, obtuse serrate leave margin and villous leaves. However, the new species differs from the latter by smaller corolla (3.2–3.5 mm), bract stalked (ca. 1.2 mm vs. sessile) and bract glabrous, except margin ciliate (Table 1, Figs 3–4). The other four species could be easily distinguished from the new species by their larger corolla (4.5–7 mm vs. 3.2–3.5 mm) and sessile bract (sessile vs. stalked ca. 1.2 mm) (Table 1, Fig. 4).

**Table 1.** Morphological comparisons among *Elsholtziazhongyangii* and its close relatives.

Species Characters		*Elsholtziazhongyangii*	*E.splendens*	E.feddeif.robusta	*E.feddeif.feddei*	*E.ciliata*	*E.souliei*
Plant	Tall	10–45 cm	30–50 cm	20–30 cm	10–20 cm	30–50 cm	< 10 cm
Inflorescence	Bract villous	Glabrous	Glabrous	Densely villous abaxially, glabrous adaxially	Villous, especially on veins abaxially, glabrous adaxially	Subglabrous abaxially, glabrous adaxially	Villous abaxially, glabrous adaxially
Flower	Corolla size	3.2–3.5 mm	6–7 mm	4.6–5.0 mm	4.5–5.3 mm	ca. 4.5 mm	ca. 6 mm
	Calyx villous	White villous outside	White hispidulous	White villous outside	White villous outside	Pilose	White villous
	Calyx apices	Apex spinescent	Caudate	Apex spinescent	Apex spinescent	Apex spinescent	Apex spinescent
	Calyx length	Two long and three short	Subequal	Two long and three short	Two long and three short	Two long and three short	Two long and three short
	Bract	Stalked, ca. 1.2 mm	Sessile	Sessile	Sessile	Sessile	Sessile
Leaves	Apices	Acute	Acuminate	Acute	Acute	Acuminate	Acuminate
	Margin	Obtuse serrate	Remotely serrate	Obtuse serrate	Obtuse serrate	Denticle	Obtuse serrate
	Blade	Villous	Sparsely fine pilose	Villous	Villous	Sparsely minutely hispid	Villous

### ﻿Molecular phylogenetics

**Figure 2.**
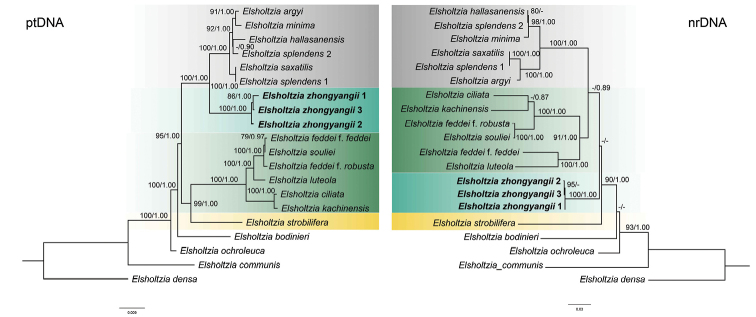
The Maximum Likelihood tree, based on the combined ptDNA (*ycf*1, *ycf*1-*rps*15, *rbc*L, *trn*L-F, *mat*K) and nrDNA (ITS, ETS) dataset without gap coding. Support values displayed on the branches follow the order ML-BS/BI-PP (“-” indicates support values of less than 75% or less than 0.85).

The aligned sequences of nrDNA (ETS, ITS) and ptDNA (*rbc*L, *mat*K, *trn*L-F, *ycf*1, *ycf*1-*rps*15) for phylogenetic analyses were 1,023 bp and 7,010 bp in length, respectively. *Elsholtziazhongyangii* was recovered as a monophyletic clade in the two resulting phylogenetic trees obtained in this study (PP: 100, BS: 1, Fig. 2). In the ptDNA tree, *E.zhongyangii* formed a sister group to the *E.argyi*-*E.splendens* clade consisting of *E.argyi*, *E.minima*, *E.hallasanensis*, *E.splendens* 1, *E.splendens* 2 and *E.saxatilis* (PP: 100, BS: 1, Fig. 2). *Elsholtziazhongyangii* and the *E.argyi*-*E.splendens* clade were sister to the *E.feddei*-*E.strobilifera* clade (PP: 95, BS: 1, Fig. 2). In the nrDNA tree, based on nrDNA, *E.zhongyangii* was sister to the E.feddeif.feddei-*E.ciliata* clade and the *E.argyi*-*E.splendens* clade, with *E.strobilifera* as one of the basal clades (Fig. 2).

Visual comparison of the resulting topologies, based on supporting values, revealed well-supported discrepancies for the combined nrDNA (ITS, ETS) and combined ptDNA (*rbc*L, *mat*K, *trn*L-F, *ycf*1, *ycf*1-*rps*15) datasets. Two trees received strong support, but with conflicting tree topologies. The *E.feddei*-*E.ciliata* clade was sister to *E.strobilifera* in the ptDNA tree (PP: 99, BS: 1, Fig. 2), but was sister to *E.argyi*-*E.splendens* clade in the nrDNA phylogeny (PP: -, BS: 0.89, Fig. 2). Due to the discrepancies between the ptDNA and nrDNA datasets, we did not combine the two matrices to further reconstruct the phylogeny.

### ﻿Taxonomic treatment

#### ﻿ Elsholtzia zhongyangii

Taxon classificationPlantaeLamialesLamiaceae

P. Li & X.J. Jin sp. nov.

372AF5AF-40F2-5F7A-AECF-45AEAD3535AD

urn:lsid:ipni.org:names:77296134-1

##### Type.

**Figure 3.**
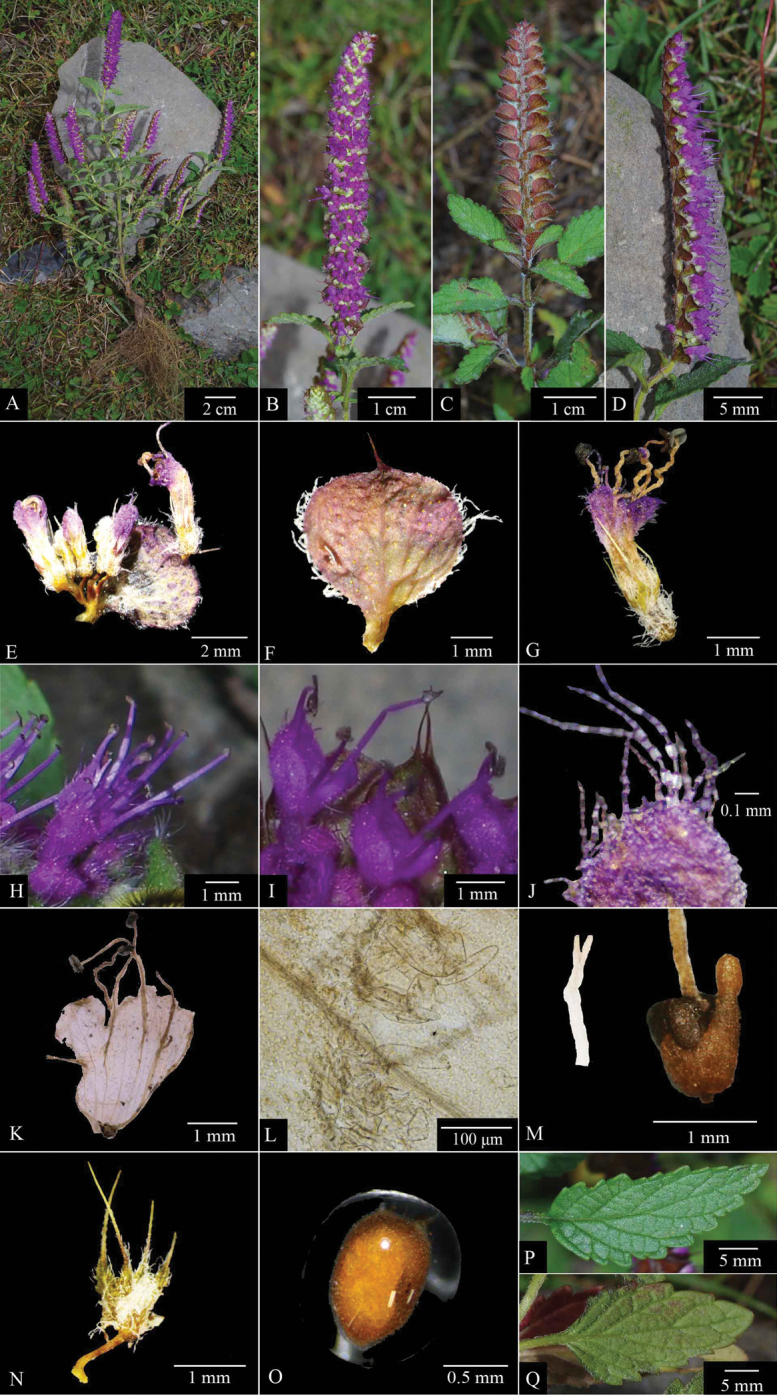
*Elsholtziazhongyangii* sp. nov. **A** blooming plant **B** front view of inflorescence **C** dorsal view of inflorescence **D** side view of inflorescence **E** cyme **F** bract **G** flower **H** side view of flower **I** front part of flower **J** villous outside corolla **K** dissected corolla **L** pilose annulate inside **M** ovary 4-cleft, 1 nectary rises on the edge of the ovary **N** outside of calyx **O** nutlet (with mucilage) **P** adaxial surface of leaf **Q** abaxial surface of leaf. Photos by Xin-Jie Jin & Pan Li.

China. Sichuan: Yajiang County, Agakeyong, 30°2.54'N, 100°15.01'E, 3237 m a.s.l., 18 Sep 2018, *Pan Li*LP185940 (holotype, ZM; isotypes CSH, CDBI, HZU, KUN, PE, SZ, WZUH). (Fig. 3 and Fig. 5)

##### Diagnosis.

*Elsholtziazhongyangii* is most similar to E.feddeif.feddei morphologically in having calyx villous, spinescent calyx apex, two long and three short calyces and acute leaf apices, but differs from the latter by its smaller corolla (3.2–3.5 mm vs. 4.5–5.3 mm), bract stalked (ca. 1.2 mm vs. sessile) and bract glabrous, except margin ciliate (vs. villous, especially on veins abaxially, glabrous adaxially).

**Figure 4.**
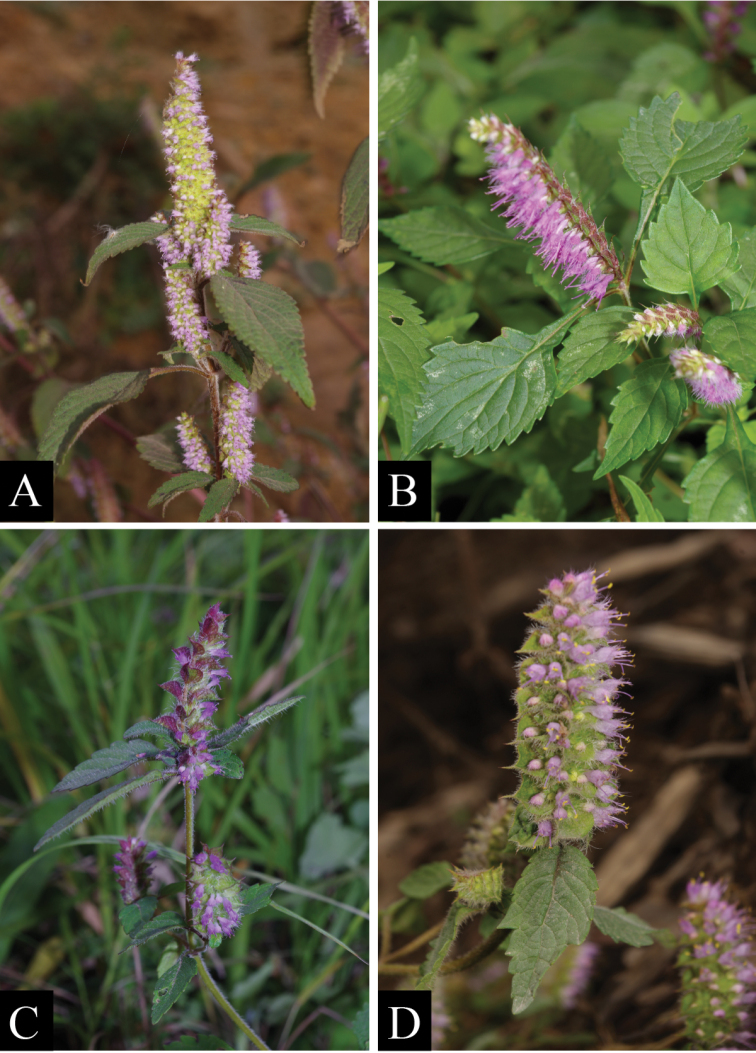
Four other sympatric or morphologically similar species **A***Elsholtziaciliata***B***Elsholtziasplendens***C**Elsholtziafeddeif.feddei**D**Elsholtziafeddeif.robusta. Photos by Pan Li.

##### Description.

Annual herbs, erect. Stems 10–45 cm tall, tawny purple; pilose or pilulose, multi-branched above base, branch apex with inflorescence, internode 0.5–4.8 cm long. Leaves ovate triangular, ovate oblong to oblong lanceolate or lanceolate, 0.7–3.6 cm long and 0.3–1.7 cm wide, apex acute, base broadly or narrowly cuneate, extending to petiole, with dense glandular. Leaves villous adaxially, villous especially on veins abaxially. Leaf margin serrate, obtuse, occasionally acute, serrate margin usually purple. Inflorescence a terminal spike, secund, ca. 0.9–7.9 cm, flowers 8–14 at each node of the inflorescence (a pair of cymes); bracts subcircular to broadly ovate, ca. 1.2–3.2 × 1.1–4.5 mm, caudate cuspidate, apex ca. 0.7–2.1 mm, glabrous, except margin ciliate, with dense glandular, dark magenta at maturity, bract stalked, ca. 1.2 mm. Pedicel ca. 2 mm long, enlarged at apex, glabrous. Calyx ca. 2.0–3.2 mm, teeth 5, teeth triangular, two long and three short, apex spinescent,white hirsute, margin ciliate. Corolla deep purple, 3.2–3.5 mm, slightly incurved, narrowly funnel-shaped, with hairs transparent and purple interlaced, villous outside, pilose annulate inside, the base of the corolla tube is about 0.5 mm wide, widening upwards, throat about 1.2 mm wide, upper lip emarginated; middle lobe of lower lip semicircular, lateral lobes subcircular, shorter than middle lobe. Stamens 4, protruding from corolla, the anterior longer than the posterior, filaments are glabrous, anthers purple-black. Style longer than stamens at maturity, exserted, 2-cleft, with lobe equal, linear. Ovary 4-cleft; disc persistent, 1 nectary rises on the edge of the ovary. Nutlets 4, brown, oblong, ca. 1.1 mm long, 0.7 mm in diameter, surface becoming mucilaginous when wetted.

**Figure 5.**
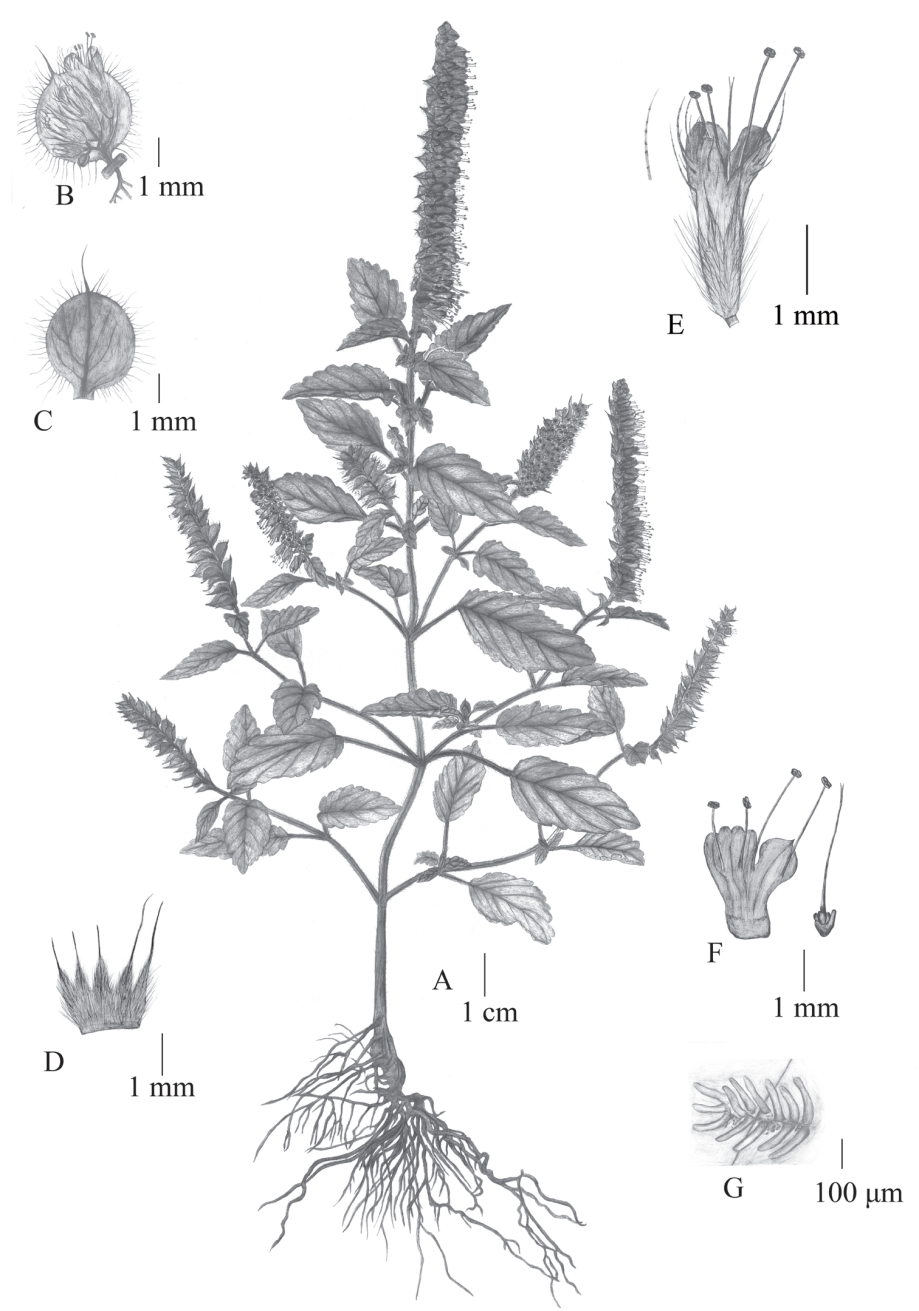
Illustration of *Elsholtziazhongyangii* Pan Li & X.J. Jin **A** the whole plant **B** cyme **C** bract **D** outside of calyx **E** flowers and villous outside corolla **F** dissected corolla **G** pilose annulate inside. Drawn by Xin-Jie Jin.

##### Phenology.

Flowering and fruiting from September to December.

##### Chinese name.

Zhong-yang-xiang-ru (钟扬香薷).

##### Etymology.

The specific epithet is named in memory of Prof. Yang Zhong, a Chinese botanist who was dedicated to botanical research and education in Xizang (Tibet), China.

##### Distribution, ecology and habitat.

– Endemic to China, Sichuan Province, Yajiang County (Wolongsi, Weidi, Agakeyong, Jialulongba and Zhusang), Litang County (Wenquan), Ganzi County, Dege County (Ezhi), Luhuo County (Zhuwo) and Batang County. Growing in grassland on mountain slopes or even along the road, at an elevation of 3000–4000 m.

##### Conservation status.

The known localities of *Elsholtziazhongyangii* are not in protected areas. During our field surveys between September 2012 and December 2021, populations in Wolongsi, Weidi, Agakeyong, Jialulongba and Zhusang of Yajiang County were found. Specimen records show that it also occurs in Litang County (Wenquan), Ganzi County, Dege County (Ezhi), Luhuo County (Zhuwo) and Batang County. Taking into consideration that it was distributed along the roadsides like weeds, we believe that it should have a much wider distribution than what is now known. Due to its wide distribution range and large population size, *Elsholtziazhongyangii* is here recommended as Least Concern (LC), according to the IUCN Categories (IUCN Standards and Petitions Subcommittee 2019).

##### Additional specimens examined (paratypes).

China. Sichuan: Yajiang County, Wolongsi, 30°2.54'N, 101°15.08'E, 3293 m a.s.l., 26 August 2015, *Pan Li & Xinglv Xie*LP150689 (PE, KUN, WZUH); Yajiang County, Weidi, 30°3.13'N, 101°12.66'E, 3028 m a.s.l., 18 September 2018, *Pan Li*LP185942 (PE, WZUH); Yajiang County, Jialulongba, 30°2.94'N, 101°18.42'E, 3760 m a.s.l., 26 August 2015, *Pan Li & Xinglv Xie*LP150686 (PE, KUN, CDBI, SZ, CSH, WZUH); Yajiang County, Zhusang, 19 September 2012, *Pan Li PNLI20120191* (WZUH); Litang County, Wenquan, 4000 m a.s.l., 11 September 1974, Y.Q. He & H.J. Wang 8281 (WUK); Ganzi County, 10 September 1951, *W.G. Hu 13099* (WUK); Dege County, Ezhi, 1900 m a.s.l., 31 August 1979, *Dege team 0685* (SM); Luhuo County, Zhuwo, 3700 m a.s.l., 2 August 1974, *Sichuan vegetation team 07693* (CDBI); Batang County, 3100 m a.s.l., 2 September 1973, *Sichuan vegetation team 3971* (CDBI).

## ﻿Discussion

*Elsholtziazhongyangii* usually grows together with *E.ciliata* or other *Elsholtzia* species in grassland on mountainsides or even along roadsides, thus it must have been overlooked previously. We found that the plant is fragrant with dense glands. This species may be suitable as an aromatic plant, and thus it has the potential for development and application values.

There is one other *Elsholtzia* species with small corolla recorded in Sichuan, i.e. *E.souliei*. However, *E.souliei* is distantly related to the new species in the phylogenetic tree (Fig. 2) and the plant is much smaller (less than 10 cm tall).

Within the genus *Elsholtzia*, the taxonomy of the *E.splendens*-*E.ciliata* clade is the most problematic. For example, in the Flora Reipublicae Popularis Sinicae, Huang (1977) divided *E.feddei* into four different forms, which looks very different from each other. However, Li and Hedge (1994) treated all the forms as synonyms of *E.feddei* in the Flora of China. A recent molecular phylogenetic study (Li et al. 2017) included two forms of *E.feddei* and showed that they did not cluster together, with E.feddeif.robusta sister to *E.souliei*. Besides, they also found that *E.splendens* from China and South Korea did not form a clade, with the Chinese *E.splendens* sister to a sympatric *E.saxatilis*, and the Korean *E.splendens* sister to *E.hallasanensis* (endemic to Jeju Island). Our phylogenetic result (Fig. 2) is consistent with previous study. Together with the extremely variable morphology and controversial taxonomic treatments, these findings imply that the so-called “*E.feddei*” and “*E.splendens*” may contain some hidden diversity. Future studies based on comprehensive sampling and genomic data are needed to shed light on the systematics of this problematic group.

## Supplementary Material

XML Treatment for Elsholtzia zhongyangii

## Supplementary materials

Supplementary material 1 Appendix I Data type COL. Explanation note Taxa and Genbank accessions involved in the present study. Missing sequences are indicated by a double slash (//). Accession numbers in bold are newly generated sequences. https://binary.pensoft.net/file/661661This dataset is made available under the Open Database License (http://opendatacommons.org/licenses/odbl/1.0/). The Open Database License (ODbL) is a license agreement intended to allow users to freely share, modify, and use this Dataset while maintaining this same freedom for others, provided that the original source and author(s) are credited.Xin-Jie Jin, Yue Huang, Yu-Kun Wei, Qing Ma, Lu-Xian Liu, Zhi-Xi Fu, Gui-Fang Wu, Yong-Hua Zhang, Pan Li
