# Supplementary material for: ﻿Elsholtziazhongyangii (Lamiaceae), a new species from Sichuan, China
Source: PhytoKeys. 2022 Mar 22;193:77–88. doi: 10.3897/phytokeys.193.80327 (PMC9849022; doi:10.3897/phytokeys.193.80327)
Supplement: Supplementary material 1 — Appendix I [file phytokeys-193-077_article-80327__-s001.docx]

**Appendix I**

**Table A1** Taxa and Genbank accessions involved in the present study. Sequences newly obtained are indicated by an asterisk (*), missing sequences are indicated by a double slash (//). Accession numbers in bold are newly generated sequences.

| **Taxon** | | | | **GenBank Accession** | | | |
| --- | --- | --- | --- | --- | --- | --- | --- |
| ***Elsholtzia*** | ITS | ETS | *ycf*1 | *ycf*1-*rps*15 | *trn*L-F | *rbc*L | *mat*K |
| *Elsholtzia argyi* | KY552491 | KY552559 | KY625121 | KY625057 | KY624990 | KY624922 | KY624855 |
| *Elsholtzia bodinieri* | KY552493 | KY552561 | KY625123 | KY625059 | KY624992 | KY624924 | KY624857 |
| *Elsholtzia ciliata* | KY552496 | KY552564 | KY625126 | KY625062 | KY624995 | KY624927 | KY624860 |
| *Elsholtzia communis* | KY552497 | KY552565 | KY625127 | KY625063 | KY624996 | KY624928 | KY624861 |
| *Elsholtzia densa* | KY552500 | KY552568 | KY625130 | KY625066 | KY624999 | KY624931 | KY624864 |
| *Elsholtzia feddei* f. *feddei* | KY552507 | KY552575 | KY625137 | KY625073 | KY625006 | KY624938 | KY624871 |
| *Elsholtzia feddei* f. *robusta* | KY552508 | KY552576 | KY625138 | KY625074 | KY625007 | KY624939 | KY624872 |
| *Elsholtzia hallasanensis* | KY552513 | KY552581 | KY625143 | KY625079 | KY625012 | KY624944 | KY624877 |
| *Elsholtzia kachinensis* | KY552514 | KY552582 | KY625144 | KY625080 | KY625013 | KY624945 | KY624878 |
| *Elsholtzia luteola* | KY552517 | KY552584 | KY625147 | KY625083 | KY625016 | KY624948 | KY624881 |
| *Elsholtzia minima* | KY552518 | KY552585 | KY625148 | KY625084 | KY625017 | KY624949 | KY624882 |
| *Elsholtzia ochroleuca* | KY552519 | KY552586 | KY625149 | KY625085 | KY625018 | KY624950 | KY624883 |
| *Elsholtzia saxatilis* | KY552525 | KY552592 | KY625155 | KY625091 | KY625024 | KY624956 | KY624889 |
| *Elsholtzia souliei* | KY552526 | KY552593 | KY625156 | // | KY625025 | KY624957 | // |
| *Elsholtzia splendens* 1 | KY552527 | KY552594 | KY625157 | KY625092 | KY625026 | KY624958 | KY624890 |
| *Elsholtzia splendens* 2 | KY552528 | KY552595 | KY625158 | KY625093 | KY625027 | KY624959 | KY624891 |
| *Elsholtzia strobilifera* | KY552531 | KY552598 | KY625161 | KY625096 | KY625030 | KY624962 | KY624894 |
| *Elsholtzia zhongyangii* 1 | KY552533 | KY552600 | KY625163 | KY625098 | KY625032 | KY624964 | KY624896 |
| *Elsholtzia zhongyangii* 2 | **OL960009** | **OL960008** | **OL960013** | **OL960014** | **OL960012** | **OL960011** | **OL960010** |
| *Elsholtzia zhongyangii* 3 | **OL960016** | **OL960015** | **OL960020** | **OL960021** | **OL960019** | **OL960018** | **OL960017** |
